# Systemic Aspergillosis in Dogs: A Historical and Current State-of-the-Art Review

**DOI:** 10.3390/vetsci13010048

**Published:** 2026-01-05

**Authors:** Talita Bordoni, Filippo Maria Dini, Roberta Galuppi

**Affiliations:** Department of Veterinary Medical Sciences, Alma Mater Studiorum-University of Bologna, Via Tolara di Sopra 50, Ozzano dell’Emilia, 40064 Bologna, Italy; talita.bordoni2@unibo.it (T.B.); filippomaria.dini@unibo.it (F.M.D.)

**Keywords:** systemic aspergillosis, dog, mycosis, *Aspergillus*, German Shepherd

## Abstract

Canine systemic aspergillosis is a rare but highly serious condition, often associated with a fatal outcome. This review encompasses all reported cases of canine systemic aspergillosis from 1978 to the present, focusing exclusively on studies in which the diagnosis was confirmed through fungal culture. A total of 155 clinical cases reported in the literature were included. Among these, the German Shepherd was the most frequently affected breed (65.16%), followed by mixed-breed dogs (7.74%). The predominant *Aspergillus* species isolated was *A. terreus* (57.69%), although other species were also reported, including *A. deflectus*, *A. fumigatus*, *A. niger*, *A. caninus*, *A. versicolor*, *A. alabamensis*, *A. citrinoterreus*, and *A. floccosus*. Recognizing clinical signs and accurately interpreting laboratory findings are crucial for early diagnosis and timely intervention, both of which can potentially improve outcomes. This review provides a detailed discussion of these aspects.

## Simple Summary

Canine systemic aspergillosis is a rare but life-threatening fungal disease with an often poor prognosis. This review covers 155 confirmed cases reported since 1978. The German Shepherd was the most involved breed and *Aspergillus terreus* was the most frequently isolated species. Early detection is difficult, making recognition of clinical signs and careful interpretation of laboratory findings essential. Key features that support timely and effective clinical decisions are highlighted.

## 1. Introduction

*Aspergillus* species are ubiquitous environmental fungi commonly found in soil, decomposing plant matter, and even in starchy foods such as cereals, potatoes, fruits, and vegetables, contributing to the deterioration of food and other products [1,2]. Although typically saprophytic, these fungi can also colonize vertebrate hosts and cause a wide spectrum of diseases. Infection usually occurs through inhalation of airborne spores, while alternative routes—including oral, urinary, transcutaneous, and transmammary exposure—have also been reported [2]. In healthy individuals, the immune system efficiently eliminates most inhaled spores, and disease is rare, reflecting the opportunistic nature of these pathogens [3].

In particular, in dogs, the most common clinical manifestation is sinonasal aspergillosis, an inflammatory condition affecting the nasal cavities and paranasal sinuses, particularly the frontal sinuses [4]. This disease may be unilateral or bilateral, and its severity depends on the virulence of the infecting strain, the host’s immune response, and the timeliness of diagnosis. *Aspergillus fumigatus* is the species most frequently involved, although *A. nidulans*, *A. niger*, and *A. flavus* have also been implicated [5,6,7,8].

Another clinical presentation is systemic aspergillosis, which, although rare, is a life-threatening condition in dogs. This form of the disease is characterized by hematogenous dissemination of *Aspergillus* spp., involving multiple organs and systems, including the respiratory, gastrointestinal, nervous, musculoskeletal, urinary, lymphatic, and ocular systems [9,10,11]. While reported cases in Europe remain limited, countries like the United States and Australia have seen more frequent reports of systemic aspergillosis [12,13]. Unfortunately, the actual prevalence of aspergillosis in dogs remains poorly understood, with many cases likely underreported [14]. This systemic form of the disease often has a poor prognosis, with a fatal outcome due to frequent relapses [15].

Early recognition of clinical signs and accurate interpretation of laboratory findings are key to achieving a timely diagnosis and intervention. In this review, these aspects are discussed by analyzing cases reported in the literature, with the aim of providing a useful tool for the veterinary clinician in managing this challenging disease.

## 2. Materials and Methods

**Figure 1:**
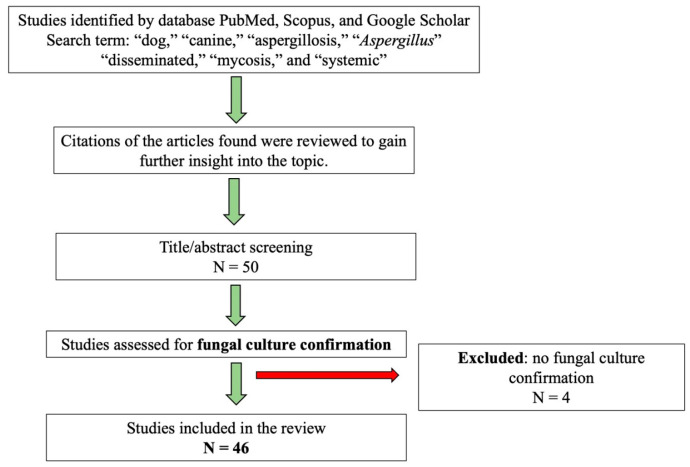
Study protocol for case selection. N = number of studies.

This review is based on data collected from advanced search engines of scholarly databases such as PubMed, Scopus, and Google Scholar, where publications were identified by searching for keywords like “dog,” “canine,” “aspergillosis,” “aspergillus,” “disseminated,” “mycosis,” and “systemic.” In addition, the reference lists of the selected articles were examined to identify further relevant studies. The search was conducted without time restriction from the earliest indexed record to the present and included articles published in English and Italian. After an initial screening, 50 articles were collected and examined; however, four studies did not perform culture-based confirmation of *Aspergillus* spp. and were therefore excluded. Consequently, 46 articles were included in the final analysis, all providing microbiological confirmation of the pathogen (Figure 1).

## 3. Results

**Figure 2:**
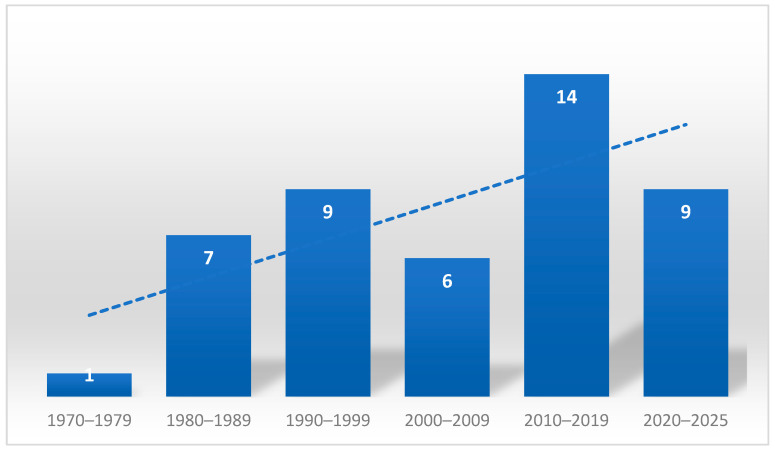
Number of publications by decade and trend line.

A total of 46 articles were included. Regarding the distribution of publications over the years, it can be observed that the first publication appeared in 1978. The number of publications on this topic shows an increasing trend in the subsequent decades, with 7 and 9 publications in the 1980s and 1990s, respectively, and a notable increase in the 2010s, reaching 14 publications. So far, in the five-year period 2020–2025, there were 9 publications (Figure 2).

### 3.1. History

The first case report of systemic aspergillosis in dogs was published in 1978 by Wood et al. [16], who described *Aspergillus terreus* infection occurring in a German Shepherd dog. Subsequently, between 1983 and 1996 in Australia and the United States [17,18,19,20,21,22], several studies were published in which German Shepherds were consistently reported as the affected breed and *Aspergillus terreus* as the only isolated species. These early reports established the association between *A. terreus* infection and German Shepherds as the classical presentation of systemic aspergillosis in dogs.

Jang et al. [23] reported four cases of systemic aspergillosis in 1986, all in German Shepherds, confirming this breed as the most commonly affected. However, in these cases, *A. deflectus*, rather than *A. terreus*, was isolated. This was one of the first reports describing other Aspergillus species as causative agents of systemic aspergillosis in dogs. Similarly, Charles [24] described a systemic case in a German Shepherd in which *A. carneus* was isolated, and Gelatt et al. [25] reported a case in the same breed with isolation of *A. fumigatus*. In 1999, Thoma et al. [26] described a classical case of *A. terreus* infection in a German Shepherd and another case in a Flat-Coated Retriever in which *A. flavus* was isolated.

In the following years, publications on this topic have become less frequent, although other *Aspergillus* species were still documented in this serious condition in dogs. Nevertheless, the German Shepherd and *A. terreus* combination remained the most frequently observed. It is important to note, however, that diagnoses from earlier years were based exclusively on conventional methods without molecular confirmation, and the true diversity of the species involved may therefore have been underestimated. More recently, reports have increased again, and several studies now include essential molecular identification.

### 3.2. Etiology

In total, from 1978 until now, 15 distinct *Aspergillus* species have been reported to cause systemic disease in dogs. *A. terreus* was responsible for 90 out of 157 (57.32%) species isolated in the cases reviewed, while *A. deflectus* caused 23 out of 157 (14.65%). *A. fumigatus* was found in 10 instances (6.36%), and *A. niger* and *A. caninus* were each detected in four cases (2.54%), *A. versicolor* in three (1.91%) and *A. alabamensis* in two (1.27%). Single cases were attributed to *A. citrinoterreus*, *A. flavipes*, *A. flavus*, *A. felis*, *A. fischerianus*, *A. puulaauensis*, *A. carneus* and *A. floccosus* (0.63%). In 13 cases (8.28%), *Aspergillus* spp. was reported without further species identification (Table 1).

When analyzing by section, the most involved were firstly the *Terrei* section (95 cases) with *A. terreus*, *A. alabamensis*, *A. citrinoterreus*, *A. floccosus*, and *A. carneus*, followed by the *Usti* section (23 cases) represented by *A. deflectus*, and the *Fumigati* section (12 cases), including *A. fumigatus*, *A. felis* and *A. fischerianus*.

Canine systemic aspergillosis is frequently sustained by *Aspergillus* species included in the *Terrei* section [9,19,27], a group that includes, beyond *A. terreus* sensu stricto (s.s.), also cryptic species such as *A. alabamensis*, *A. allahabadii*, *A. ambiguus*, *A. aureoterreus*, *A. carneus*, *A. hortai*, *A. microcysticus*, *A. neoafricanus*, *A. neoindicus*, *A. niveus*, *A. citrinoterreus*, *A. bicephalus*, *A. iranicus*, *A. floccosus* and *A. pseudoterreus* [28]. It is evident from the literature that *A. terreus* is the most involved species, although many studies have not conducted additional molecular studies to distinguish species within the *Terrei* section, as they are often morphologically identical. Lim et al. [27] report that dogs infected with other species of *Aspergillus* showed better survival rates than those affected by *A. terreus*, suggesting that the *A. terreus* complex may be more pathogenic than other *Aspergillus* species. The involvement of this species in systemic forms may be linked to its ability to produce aleurioconidia, structures that increase its capacity for hematogenous spread, and consequently, its pathogenicity [28].

**Table 1:** Reported distribution of *Aspergillus* species based on 156 isolates. The most frequently identified species was *A. terreus*; 13 isolates remained unidentified at the species level.

Aspergillus Species	No. of Isolates	References
*A. terreus*	90	[4,9,13,14,15,16,17,18,19,20,21,22,26,27,29,30,31,32,33,34,35,36,37]
*A. deflectus*	23	[9,23,27,38,39,40,41]
*A. fumigatus*	10	[9,15,25,27,34,37,42,43]
*A. niger*	4	[9,43,44]
*A. caninus*	4	[45,46,47,48]
*A. versicolor*	3	[15,49,50]
*A. floccosus*	1	[51]
*A. alabamensis*	2	[27,52]
*A. citrinoterreus*	1	[53]
*A. flavipes*	1	[9]
*A. fischerianus*	1	[40]
*A. puulaauensis*	1	[27]
*A. flavus*	1	[26]
*A. felis*	1	[54]
*A. carneus*	1	[24]
*Not identified*	13	[15,18,29,34,37,55,56,57]
Total isolates	157	

### 3.3. Geographic Distribution

**Figure 3:**
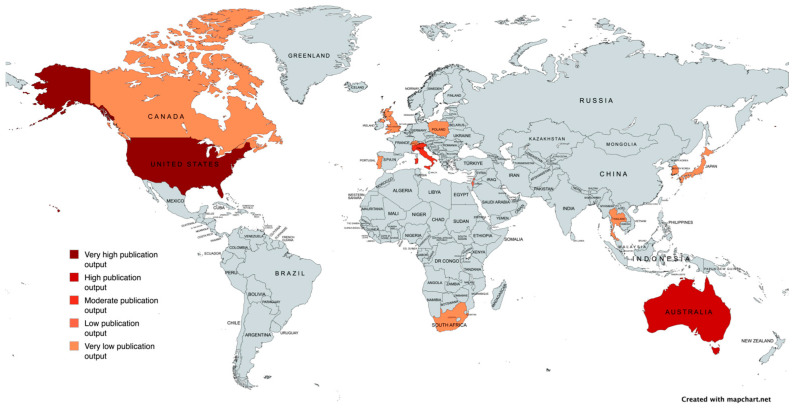
Geographical map of reported cases of canine systemic Aspergillosis, with color intensity reflecting case frequency. Created with mapchart.net.

Hot and humid climates may serve as risk factors for systemic aspergillosis [32]. Furthermore, areas near construction sites could pose a risk to humans [58], and it is possible that dogs, which share many environments with humans, could also be affected. In fact, Patterson et al. [58] recommend minimizing the exposure of at-risk patients to construction and renovation activities. Cases reported in the literature have been mainly published in the USA, with 19 publications (41.30%) [9,15,16,17,20,23,25,29,30,32,34,38,45,49,52,53,55,56,57], followed by Australia with 11 publications (23.91%) [18,19,21,24,27,31,39,40,41,48,54]. Furthermore, cases have also been reported in Italy (10.86%) [12,13,37,42,51] and Israel (4.34%) [4,14], and one case (2.17%) was reported by each of the following states: Canada [46], Japan [47], Poland [43], Portugal [36], South Africa [23], South Korea [50], Thailand [44], the United Kingdom [35], and Switzerland [26] (Table 2, Figure 3).

Given that most publications originate from the USA and Australia, the observed geographic distribution of cases may reflect research activity rather than true environmental patterns. Moreover, the presence of *A. terreus* clusters in geographically distant regions such as Tyrol (Austria) and Texas (USA) (two areas that do not share a common climate or environmental background) further supports the hypothesis that local climatic conditions may not be the key drivers of this unusually high prevalence, suggesting that multiple factors, including host-related characteristics, may contribute to this phenomenon [59].

### 3.4. Breed, Age, Sex Distribution and Predisposing Factors

The analyzed data clearly show that German Shepherd (GS) dogs are significantly overrepresented, accounting for 101 out of 156 cases (64.74%) [4,9,12,15,16,17,18,19,20,21,22,23,24,25,26,27,29,32,33,34,37,39,40,41,49,51,55,56]. Additionally, mixed-breed dogs were reported in 12 out of 156 cases (7.69%) [9,13,15,27,36,37,46,53], while other specific breeds included Labrador Retrievers (5, 3.20%) [9,30,44], Vizsla (3, 1.92%) [15,16,17,18,19,20,21,22,23,24,25,26,27], Pugs (2, 1.28%) [9,35], Springer Spaniels (2, 1.28%) [38,52], and Rhodesian Ridgebacks (2, 1.28%) [9]. A single case each was reported for the following breeds (0.64%): Kurzhaar dog [42], Newfoundland [15], Giant Schnauzer [15], Miniature Schnauzer [57], Shiba Inu [47], Saint Bernard [43], Cocker Spaniel [45], Rottweiler [48], Coton de Tulear, Dalmatian [50], Red Cloud Kelpie [21], English Setter [9], Whippet [9], Flat Coated Retriever [26] and Bull Terrier [37]. In 10 cases, the breed was not available (9%) (Table 3).

**Figure 4:**
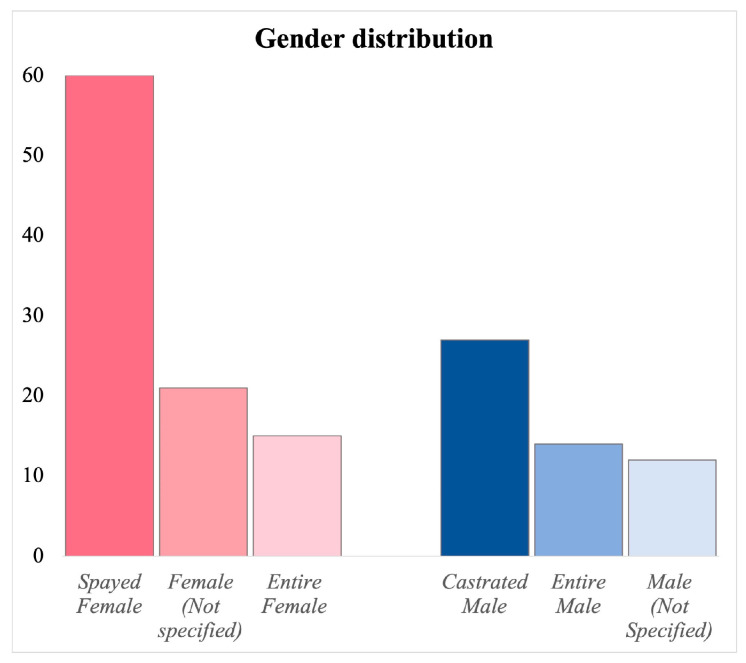
Distribution of gender across case reports, showing the number of intact and sterilized females and intact and castrated males and the cases in which this type of information was not specified.

In terms of gender distribution, females are more frequently affected by this condition. Specifically, 96 females were included (61.53%), with 15 being entire and 60 spayed, and for 21 females, it was not specified whether they were entire or spayed. On the male side, there were 54 cases (34.61%), including 15 entire and 27 neutered, and in 12 cases, it was not specified whether they were entire or neutered. In 6 cases (3.84%), this type of information was not available (Figure 4).

The included cases had a mean age of 4.4 years, with a median age of 4 years and a standard deviation of 2.5.

Among the predisposing factors, we must consider breed predisposition, the presence of concomitant diseases, and corticosteroid, antibiotic, or chemotherapy treatments. The predisposition of German Shepherds to develop this disease seems to be attributed to a hereditary primary immune deficiency related to a mucosal immunity deficit. Comparative studies on immunoglobulin concentrations in healthy dogs indicate that the IgA levels in German Shepherds tend to be lower than those found in other breeds [11,41,49]. Nevertheless, other breeds can also be affected by systemic aspergillosis, especially if there is an underlying immunosuppressive condition related to disease and/or therapies [52]. These therapies were frequently administered in the reported cases and may have increased the patients’ susceptibility to the disease. In particular, Lim et al. [27] describe that, among 34 dogs with systemic aspergillosis, 16 were treated with antibiotics and 8 with glucocorticoids. Other authors [29,38,39,41,45,52], report that, at the initial presentation, veterinarians almost always start treatment with antibiotics or corticosteroids. Only after a few days, once a fungal infection is confirmed by cultural methods, antifungal therapy is initiated. This represents a major challenge in fungal diseases, as the time required for culture often delays diagnosis and appropriate treatment. Dogs with an underlying predisposing condition are at particular risk. In three cases described in the literature [45,53,54], dogs presumably affected by immune-mediated hemolytic anemia, previously treated with immunosuppressive therapy, developed systemic aspergillosis. These observations highlight the importance of carefully evaluating the patient’s medical history, breed predisposition, and any underlying diseases or immunosuppressive conditions.

### 3.5. Pathogenesis

The pathogenesis of systemic aspergillosis can vary across hosts and clinical contexts. The entry route is not always identified in natural cases, as at the moment of diagnosis, the fungus has already spread to various organs. However, by evaluating the organs primarily and predominantly affected, it is possible to hypothesize the route of penetration [12]. Generally, this is represented by the inhalation route and, as in rhinosinusal aspergillosis, conidia in the environment can be inhaled and settle at the level of bronchioles and alveoli [52]. In healthy individuals, these conidia are rapidly cleared, but in the case of immunocompromised patients, conidia can germinate and develop filamentous hyphae [60,61]. The mycelium that develops can then give rise to pulmonary or bronchopulmonary infection. From the pulmonary site, hematogenous spread of spores can occur, which can result, as anticipated earlier, in fungal infection of multiple organs [52]. It should be emphasized that *A. terreus* is capable of producing aleurioconidia [29]. These accessory spores produced directly by fungal hyphae in infected tissues can easily be released into the bloodstream following vessel penetration by the hyphae. The aleurioconidia trigger a very intense inflammatory response in the tissues. In addition, A. terreus also produces various special enzymes and secondary metabolites, such as asp-melanin, which result in alterations in the immune system, also contributing to the spread of the fungus. Due to the presence of these virulence factors, *A. terreus* exhibits a particularly aggressive behavior compared to other species of its genus, which allows it more easily to cause severe and disseminated infections [62]. The virulence profile of an isolate of *A. terreus* responsible for aspergillary osteomyelitis revealed the production of enzymes that increase its pathogenicity, including lipase, hemolysin, and DNAase [36]. In fact, these enzymes can impair cell function, causing cell lysis and consequently leading to tissue necrosis. In addition to spore inhalation, Aspergillus spp. can enter the host through other routes, which can always be followed by hematogenous spread [52]. In fact, in some cases reported in the literature, injuries to the respiratory system are not reported, suggesting a different portal of entry into the host. Depending on the clinical cases and injuries reported, penetration through the gastrointestinal tract, the ascending urinary route and the transcutaneous route through wounds is considered probable [12,36]. A case of systemic aspergillosis has also been described in a bitch after mating [56]. Considering that Siemieniuch et al. [43] found hyphae in the semen of a male dog that had mated in the preceding months, it remains plausible that sexual transmission could represent an additional potential route of entry. Supporting this hypothesis is the presence of several cases of pyometra reported in the literature [18,32,55], as well as a peculiar case of transuterine transmission from a pregnant dog to the puppies born already infected [33].

After entering the host, at the tissue level, the fungal hyphae and the enzymes produced cause a necrotizing effect and result in the onset of granulomatous or pyogranulomatous inflammation to which dysfunction of the affected organs follows [11].

Furthermore, *A. fumigatus* is able to cross the pulmonary epithelium and can cause localized or systemic infections [63]. Its invasion and spread through the body, in fact, can occur in different ways, as described by the authors. Aspergillus fumigatus usually crosses the pulmonary epithelium [64] and then the adhesion is mediated by fungal molecules, such as sialic acid, galactosaminogalactan and β-1,3 glucan [65,66,67]. After attachment, A. fumigatus is incorporated into epithelial cells through actin-dependent endocytosis [68,69,70,71] and itself produces substances that help it enter more efficiently [71,72,73]. Once inside the cells, some of the conidia are killed, but about 3% remain viable [68]. Of these, one-third germinate and form hyphae that grow and invade lung tissues and blood vessels, without destroying host cells [68,70,74]. It is also hypothesized that *A. fumigatus* may use phagocytes as a vehicle to escape from the lungs. *Aspergillus fumigatus*, in fact, can survive inside these phagocytes [75,76]. In addition, some dendritic cells can transport the fungus to mediastinal lymph nodes (where pathogens are filtered out) [77], from which it could spread into the bloodstream and infect other organs, such as the liver, kidneys, spleen and brain [78]. The precise mechanism by which *A. fumigatus* crosses the blood–brain barrier (BBB), which protects the brain, is not yet well understood, but it has been suggested that gliotoxin may damage the endothelium and promote transmigration of the fungus [79]. In human medicine, these pathogenic mechanisms have been extensively investigated and characterized through numerous experimental studies, in particular with *A. fumigatus* strains. By contrast, veterinary literature on this topic is still scarce. Nevertheless, it is reasonable to assume that the pathogenetic mechanisms in animals are largely comparable to those described in humans. In veterinary medicine, the most common sites of fungal dissemination are the kidneys, intervertebral discs, central nervous system, eyes, lungs, and long bones [13]. Nevertheless, necropsy studies have revealed that the fungus can also colonize a wide range of organs, such as the brain [4,9,17,20,23,24,32,34], heart [4,9,16,17,18,19,20,22,25,38,39,52], spleen [4,9,16,17,18,19,20,21,22,23,24,25,26,32,39,52,55], pancreas [9,18,20,23], liver [9,16,17,18,20,23,25,38,52], bone marrow [9,18,19], lymph nodes [4,9,13,18,19,20,22,23,32,34,38,39], uterus [18,32,33], adrenal glands [38,39], diaphragm [20,25,34,52], gallbladder [52], small intestine [9,23], skin, prostate, trachea and larynx [9]. This fact shows how the fungus is able to colonize any type of organ system in the affected host and highlights the need to consider multiple organ systems when facing diagnostic hypotheses of canine systemic aspergillosis.

### 3.6. Clinical Signs

Clinical signs related to mycoses can vary widely in both severity and location, with manifestations developing either suddenly or chronically, as observed in numerous studies [41,49]. Initially, nonspecific symptoms are usually observed, which may include fever, weakness, weight loss, and lymphadenomegaly, progressively worsening as the disease progresses. Data obtained from the cases reviewed confirm that these nonspecific signs are common, with fever, weakness, lethargy, and weight loss among the most frequent. Other nonspecific signs, such as anorexia and loss of appetite, are also present, although in fewer numbers.

Of interest, in some cases, the initial symptoms may be localized, with nonspecific systemic signs appearing only later [13]. This suggests that mycosis may initially present as a localized infection and subsequently progress to a more generalized form, affecting multiple body systems. Once the infection disseminates, clinical signs related to specific organs or tissues emerge, with the nervous system being among the most frequently affected. In the cases analyzed, neurological signs were observed in a substantial number of patients, including spinal pain (back or neck pain) and paresis or paralysis in many subjects. Other notable neurological signs included ataxia and vestibular abnormalities, such as head tilt, nystagmus, and circling. Additionally, the dogs described in the reviewed articles exhibited, upon examination, decreased postural reactions, seizures, paraspinal hyperesthesia, absence of the menace reflex, anisocoria, proprioceptive deficits, right facial paralysis, hyperactive patellar reflexes bilaterally, mental dullness and depression, and miosis.

Musculoskeletal involvement is another common manifestation, with lameness often being one of the first clinical signs observed, frequently accompanied by joint swelling. The occurrence of pathological fractures and muscle atrophy further indicates a direct impact on bone and muscle structures, likely related to osteomyelitis or joint infection. These orthopedic signs are frequently accompanied by motor difficulties, such as reluctance to walk or jump, reflecting impaired motor function.

In addition, the gastrointestinal system may be affected, with clinical manifestations including vomiting, diarrhea, hepatosplenomegaly on palpation, and, in some cases, hematochezia or melena [30]. Oral lesions, such as ulcers, have also been reported [23], further illustrating the potential for Aspergillus to directly involve multiple visceral and mucosal sites. Ocular involvement is also common, with clinical signs including uveitis, retinitis/chorioretinitis, panophthalmitis, hyphema, exophthalmos, scleral injection, rubeosis, ocular discharge, conjunctivitis, and vitreous opacity, which may lead to impaired vision. Severe inflammation may manifest as intraocular hypertension or exophthalmos, potentially resulting in permanent ocular damage if not treated promptly.

Clinical signs attributable to a problem of the uro-genital system were present in several cases, with polyuria/polydipsia, pyometra, serosanguinous vulvar discharge, and other problems such as cystitis and kidney injury, which are compatible with a functional impairment of the kidneys or lower urinary tract. The presence of *Aspergillus* spp. in the reproductive tract is an aspect that has not yet been fully explored. This is an important factor to consider, together with all the clinical, diagnostic, and reproductive implications of such a localization. Over the years, different authors have reported the presence of *Aspergillus* spp. in the uterus of both pregnant and non-pregnant females. Examples include the case reported by [56], which describes aspergillosis occurring after mating. Furthermore, a case description is also available of a pregnant infected female that gave birth to infected puppies, representing the first report of transuterine transmission of aspergillosis [33]. Cases of pyometra have also been documented by other authors, including [18,32,55]. While *Aspergillus* infection is well-documented in the female reproductive tract, similar involvement is also reported in males. For example, Siemieniuch et al. [43] identified fungal hyphae in the semen of a breeding male dog after multiple matings. Additionally, Jang et al. [23] described scrotal swelling among the clinical signs, and Wilson and Odeon [30] reported cases with an enlarged prostate, demonstrating that Aspergillus can also target the male reproductive system.

The respiratory system, being one of the main sites of entry of this fungus, can also be affected. In the cases reviewed in the literature, the most frequent symptom is cough, followed by respiratory distress, but tachypnea, epistaxis, crackles, mucoid and/or serosanguinous nasal discharge and decreased respiratory rate have also been reported.

Cardiac signs are relatively uncommon, but those that have been reported include heart murmurs, arrhythmias, tachycardia, bradycardia, and hypertension.

In addition, dermatological signs, including skin lesions, a dull hair coat, and pruritus, have also been documented, although it is unclear whether these manifestations are directly related to Aspergillus infection or secondary to underlying conditions. In Table 4, the clinical signs involving the different organs or organ systems are summarized in order of frequency.

In summary, the variability of clinical signs observed in the cases analyzed underscores the complexity of this mycosis and its ability to involve multiple organs. Although nonspecific symptoms are most common in the early stages, involvement of specific systems, such as neurological, musculoskeletal and ocular, may indicate severe disease progression, requiring timely and targeted treatment. Knowledge of these signs and their evolution is crucial for early diagnosis and appropriate management of mycosis.

### 3.7. Diagnosis

The diagnosis of systemic aspergillosis is complex and is often reached late when the clinical picture is advanced. The reasons are related to the rarity of the disease and the fact that it manifests as varied and nonspecific clinical signs. Moreover, fungal etiology is often considered only after other differential diagnoses have been ruled out, and this contributes to the failure of therapy [42]. Therefore, it is important to be aware of this deep mycosis and include it among the differential diagnoses, especially in individuals with risk factors, such as German Shepherd breed dogs or patients with immunosuppression [9]. The diagnostic procedure varies depending on the nature of the symptoms present, in each case including laboratory investigations of blood or urine to assess the patient’s clinical picture, instrumental investigations and tests aimed at identifying the etiologic agent [42].

#### 3.7.1. Hematology, Biochemistry and Urinalysis

In general, hematological and biochemical abnormalities in canine systemic aspergillosis reflect the inflammatory process and the organs involved rather than providing specific indications of the infective agent. The most commonly reported hematologic alterations include neutrophilia, leukocytosis, the presence of toxic neutrophils, and normocytic, normochromic anemia associated with chronic kidney disease [9,11]. In the cases analyzed, additional hematologic changes included monocytosis, eosinophilia, lymphopenia, lymphocytosis, thrombocytosis, and thrombocytopenia.

Biochemical abnormalities frequently observed comprise hyperglobulinemia and markers of renal involvement, such as increased serum creatinine, urea, and calcium, often accompanied by hypoalbuminemia [11]. Notably, elevated serum creatinine at diagnosis has been correlated with a poorer prognosis, suggesting that azotemia may serve as a negative prognostic indicator in systemic aspergillosis [27]. In the analyzed cases, the most frequent biochemical derangements were azotemia, hyperglobulinemia, increased Alkaline Phosphatase (ALP), increased total serum protein, hypoalbuminemia, elevated Alanine Aminotransferase (ALT) and Aspartate Aminotransferase (AST), increased C-Reactive Protein (CRP), hyperphosphatemia, hyperbilirubinemia, hypokalemia, hyperfibrinogenemia, increased amylase and lactate dehydrogenase, hypochloremia, hypoglycemia, hypercalcemia, increased Creatine Kinase (CK) and Gamma-Glutamyl Transferase (GGT), and hyperglycemia.

Urinalysis often reveals isosthenuria or hyposthenuria, hematuria, and pyuria [11]. In the reviewed cases, additional urinary abnormalities included proteinuria, the presence of leukocytes, and bilirubinuria. Importantly, in cases with renal involvement, fungal hyphae may be detected in the urine sediment, providing a rapid and noninvasive diagnostic tool that was utilized in several cases included in this review (Table 5).

#### 3.7.2. Diagnostic Imaging

Even though blood tests can serve as an important initial screening tool in suspected cases, diagnostic imaging also represents a key step in the diagnostic process. Indeed, radiography, ultrasonography, computed tomography (CT) and magnetic resonance imaging (MRI) can often reveal significant pathological alterations that support the diagnosis. Radiography is considered a first-step diagnostic imaging approach: it is non-invasive, relatively inexpensive, and feasible in almost all veterinary clinics [80]. However, it is not able to detect certain abnormalities that can instead be identified using more advanced imaging techniques such as CT or MRI. In the clinical cases examined, imaging allowed the objective identification of several pathological changes, including orthopedic, neurological, gastroenteric, respiratory, and urogenital disorders. Radiography and CT of the axial and appendicular skeleton were particularly valuable in cases involving orthopedic problems. The most common findings included areas of bone lysis, pathological fractures, marked periosteal reactions, and vertebral subluxations associated with osteomyelitis and discospondylitis. Osteomyelitis most frequently affects long bones, although it may also involve other bones such as the vertebrae or sternebrae [35]. Del Magno et al. [13] described a case of *Aspergillus* osteomyelitis affecting the wing of the ilium, while Brocal et al. [35] documented osteolysis of the petrous part of the temporal bone in a Pug with systemic aspergillosis and an evident head tilt. Radiography also allows evaluation of the lungs and thoracic cavity, an essential procedure if respiratory symptoms are present. Pulmonary fields may show areas of increased radiopacity, often with interstitial or alveolar patterns consistent with inflammatory lesions caused by fungal infection [12,42]. In the cases reviewed, imaging revealed increased airway and vascular density, pulmonary involvement, and cardiomegaly. A common radiographic finding is thoracic lymph node enlargement and, in some cases, pleural effusion [9,12,38] or mediastinal masses were also observed [9,39,44,46,50]. Additionally, imaging revealed uterine and renal enlargement [18] as well as lymphadenomegaly in multiple anatomical regions.

At the abdominal level, ultrasonography is the primary diagnostic imaging technique used. The most common findings in cases of systemic aspergillosis include enlargement of the abdominal lymph nodes, peritoneal effusion, and abnormalities involving the kidneys, spleen, liver, stomach, and pancreas [9,11,12]. At the renal level, ultrasonography may reveal pelvic ectasia with echogenic debris within the renal pelvis, architectural distortion of the organ, hydronephrosis, and the presence of nodular lesions, often consistent with pyelonephritis [9].

Several studies have employed advanced imaging techniques, specifically computed tomography (CT) and magnetic resonance imaging (MRI). In particular, CT represents an excellent tool for detecting several alterations, such as discospondylitis [37], enlargement of thoracic and abdominal lymph nodes or other forms of lymphadenopathy [9,44,47,48,50], as well as other multiorgan alterations, including hepatomegaly, splenomegaly, and changes in the lungs and kidneys [41,48].

MRI, which is particularly suited for studying the nervous system, has been utilized by several authors. Taylor et al. [34] and Schultz et al. [9] observed brain alterations using this sensitive technique. Moreover, cases of discospondylitis visualized via MRI have also been reported [34,37,51]. Finally, Brocal et al. [35] and Del Magno et al. [13] described bone lysis associated with surrounding muscular lesions.

Discospondylitis, the most frequently observed imaging abnormality in aspergillosis, is an infection of the intervertebral disc and adjacent vertebrae caused by bacteria or fungi that reach this site via the bloodstream or secondary to previous trauma or surgery [37]. It represents one of the most frequently reported clinical manifestations in cases of aspergillosis. In the reviewed studies, a considerable number of cases have been described. Analysis of the various localizations indicates that the thoracic region is the most commonly affected, followed by the lumbar region. The cervical and sacral regions are less frequently involved, specifically for systemic aspergillosis (Table 6).

Although the most prevalent skeletal alterations are observed in the axial skeleton, the literature also reports additional sites of bone injury. In particular, regarding the long bones, abnormalities of the humerus are described [9,18,36,39,49]. The tibia was affected in two reports [9,23], and femoral involvement has also been described [21]. Lesions of the left elbow [4,38] and carpal involvement together with hock lesions are also documented [18]. The scapula was affected in two reports [9,18].

Furthermore, other less frequently involved sites include the ribs [9,20], the fifth metacarpal [21], the wing of the ilium [13], and the sternebrae and sternum [9,49].

All the diagnostic imaging findings are summarized in Table 7.

#### 3.7.3. Mycological Examination

Pathological tissues can be used to obtain samples for cytological, histological, and cultural examinations, which are essential for reaching a definitive diagnosis of systemic mycosis or systemic aspergillosis. Often, molecular biology techniques are required to accurately identify the fungal species involved [41]. Depending on the anatomical site and the type of examination to be performed, samples can be obtained through more or less invasive methods. Cytology and histology are essential tools for the definitive diagnosis of mycoses. In particular, cytology represents a rapid and highly indicative method. Several authors have utilized this initial diagnostic tool to guide the diagnostic process toward a suspected mycosis [13,35,41,44,46,52,53]. Cytological analysis of pathological tissues can be performed on minimally invasive or non-invasive samples, such as fine-needle aspirates or abdominal and thoracic fluid samples. Fine-needle aspirates of peripheral lymph nodes affected by lymphadenopathy have particular diagnostic value, as they represent a simple method for achieving a diagnosis of systemic mycosis. The main cytological findings typically include the presence of inflammatory cells, severe chronic inflammation, and fungal elements. When a surgical procedure is necessary, cytology can also be performed intraoperatively, providing the surgeon with real-time information that can guide surgical decisions. This approach also allows early initiation of broad-spectrum antifungal therapy, while awaiting biopsy, culture, and molecular results that can confirm or adjust the treatment plan [35].

For histopathological examination, biopsies obtained during procedures are required; for example, bone or intervertebral disc biopsies are particularly challenging [11,12]. Both histological and cytological preparations allow visualization of fungal hyphae. Their presence is particularly evident when using specific staining such as Periodic Acid-Schiff (PAS) or Grocott–Gömöri’s methenamine silver stain (GMS) [12], and the hyphae are often surrounded by pyogranulomatous inflammation [11,12,13].

Fungal cultures, which are crucial for a correct identification of the pathogen, can be obtained from body fluids or other affected tissues [9,11] (Table 8). Lymph node aspirates, as well as urine, can also be used [13]. In 65 clinical cases analyzed, urine culture was used for the isolation of *Aspergillus* spp. It is essential to be familiar with this method and to prioritize its use, as it is non-invasive, simple, and rapid. Interestingly, the finding of hyphae in the vitreous humor suggests that this kind of sample can be effective for an accurate diagnosis when ocular symptoms are present [19,25].

#### 3.7.4. The Role of Molecular Diagnostics

Over the years, molecular biology has been used to diagnose *Aspergillus* species more reliably and accurately. The nuclear rDNA internal transcribed spacer region (ITS1-5.8S-ITS2) serves as the official DNA barcode for fungi [81]. Additionally, for *Aspergillus* species, three more specific markers are available: calmodulin (CaM), β-tubulin (BenA), and the second largest subunit of RNA polymerase II (RPB2) [82]. Nowadays, this approach is of fundamental importance for supporting classical diagnostic methods. In fact, many species of *Aspergillus* belonging to the same section have very similar characteristics and risk being confused, which is why most diagnoses made without molecular support could actually involve other species belonging to the same section. Furthermore, there are cases in which the morphology was not at all indicative; some strains showed slow growth and phenotypic atypia compared to classical *Aspergillus* strains [41,52]. Similarly, in a reported case of systemic aspergillosis due to *Aspergillus floccosus*, species-level identification was achievable only through molecular methods, as both microscopic and macroscopic features displayed atypical characteristics [51]. Also, Sender et al. [53] emphasize the critical role of molecular confirmation in accurately identifying species within the *Terrei* section. In their study, the isolate was initially classified as *A. terreus* based on conventional laboratory findings; however, molecular analysis subsequently revealed the organism to be *A. citrinoterreus*, a distinct species within the same section. All these findings underscore that, while conventional diagnostic approaches have historically provided valuable insights, molecular methods are now indispensable to achieve accurate species identification. Focusing on the *Terrei* section isolates, various authors [12,36,51,53] confirmed their identification with molecular methods. Other authors [45,46,47,48] used these methods to confirm the isolation of *Aspergillus caninus*. Similarly, an atypical isolate of *A. deflectus* was confirmed through DNA amplification and sequencing of its culture [41]. Molecular confirmation was also performed for another case of systemic aspergillosis by *A. deflectus* [39] and for two cases caused by *A. versicolor* [49,50].

#### 3.7.5. Serological Test

Serological testing for anti-*Aspergillus* antibodies using agar gel immunodiffusion is not reliable for the diagnosis of canine systemic aspergillosis [9,10]. A non-invasive, sensitive, and specific alternative is the detection of *Aspergillus* galactomannan antigen (GMA) in body fluids via ELISA in serum or urine. Galactomannan is released by the fungus into body fluids, and the test quantifies its concentration by measuring optical density. False positives may occur due to other systemic mycoses or during treatment with fluid replacement (Plasmalyte) or beta lactam antibiotics [14,15,34], while false negatives are reported in localized pulmonary or sino-nasal aspergillosis, where GMA levels are low. The GMA ELISA applied to serum and urine shows a sensitivity of 93 and 89%, respectively, when compared with the fungal cultures [10]. In another study, the test for serum galactomannan was positive in 2 out of 5 dogs affected by systemic aspergillosis [27], while [34] reported that this test was positive in all three cases tested. According to [10], in cases of disseminated disease, GMI values are very high and may correlate with disease severity, potentially serving as a prognostic marker in dogs. In addition, considering the generally poor prognosis associated with this disease and the time needed for a fungal culture, this test could serve to identify the infection and start the treatment earlier. However, further studies are needed to determine whether *Aspergillus* GMA results can be used to assess treatment response and clinical relapse in dogs with systemic aspergillosis [15].

### 3.8. Necropsy Findings

In a lot of the cases included in this review, necropsy data are available, with a description of the organs’ lesions. In particular, macroscopically, multiple nodules and plaques can be detected in several organs after the progression of systemic aspergillosis. They can usually be multiple and miliary, but also multifocal to coalescing or forming firm plaques and nodules. The color can vary between white-yellow-brown. On cross section, these foci often had a necrotic center. The affected organs included the brain, heart, lungs, pleura, trachea/larynx, diaphragm, liver, pancreas, stomach, small intestine, spleen, lymph nodes, kidneys, urinary bladder, uterus, testis, prostate, skeletal tissue, bone marrow, joints, eyes and peritoneum (Table 9). In addition to this classical finding with nodules and plaques, red-brown effusion in the pleural, peritoneal, and pericardial spaces is also reported [52], as well as hemorrhagic effusion within the abdominal cavity [29].

### 3.9. Therapy and Outcome

The management and treatment of canine aspergillosis appear highly variable across the literature, largely reflecting differences in drug availability, owner finances and compliance, adverse effects, and the absence of standardized treatment guidelines. From published cases, it emerges that itraconazole remains the most frequently used, although its efficacy is inconsistent and prolonged administration is often required [21,27,56]. Several reports describe cases in which itraconazole was selected because of financial problems of the owners or intolerance to other azoles. However, when the treatment was discontinued, whether due to cost or gastrointestinal side effects, clinical deterioration often occurred [13,47].

First-generation azoles, such as ketoconazole, consistently show poor clinical outcomes, with multiple accounts of absent improvement or rapid deterioration [20,22,28]. Fluconazole, although used in some cases, appears ineffective in vitro and in vivo, often prompting a switch to itraconazole or newer agents once sensitivity testing becomes available [35,47,51,56].

Voriconazole, posaconazole, and terbinafine emerge as valuable options, particularly in refractory infections or when susceptibility testing indicates resistance to itraconazole. Several multimodal regimens, including combinations of itraconazole with voriconazole, terbinafine, or amphotericin B, have yielded prolonged survival times or meaningful clinical improvement [9,27,34].

Survival outcomes vary widely, from very short times in untreated or ketoconazole-treated cases to exceptionally long survival in dogs receiving prolonged treatment with itraconazole or other multimodal therapy.

Notably, a survival of 6.5 years with aggressive multimodal therapy is reported [27], highlighting the potential benefit of combined pharmacologic and surgical approaches. However, amphotericin B frequently induces azotemia, which causes early discontinuation and often coincides with clinical deterioration [44,45].

Two cases with long-term survival are available in earlier literature [21]. The first dog survived for 1095 days while receiving continuous itraconazole therapy; after the treatment became inconsistent, it survived for an additional 485 days. Similarly, the second dog survived for 1000 days on itraconazole and then for only 572 days after the therapy became irregular, highlighting the importance of maintaining consistent treatment. In the case series by Okonji et al. [37], a patient treated with itraconazole was alive after 1130 days. In contrast, Bennett et al. [41] reported a case with 1151 days of survival without any treatment, underscoring the unpredictable natural history of the disease. Studies using posaconazole showed partial responses and occasional long-term remission, though relapses were common when therapy became inconsistent [15].

Overall, the literature suggests that while itraconazole remains the most accessible and commonly used antifungal, multimodal therapy involving newer triazoles, terbinafine, and/or surgical debridement tends to yield longer survival times. Nevertheless, prohibitive costs and adverse effects limit the consistent use of advanced antifungals, contributing to the highly variable outcomes of this disease.

## 4. Discussion

Dog systemic aspergillosis is a rare condition with few descriptions in the literature all over the world, with the German Shepherd dog being the most frequently affected breed. Nevertheless, relying solely on the published literature may result in a bias concerning the true occurrence of the disease. The cases included in this review come primarily from university hospitals or specialty clinics and therefore may not represent the full population of dogs with systemic aspergillosis. It is indeed important to consider that the diagnosis of dog systemic aspergillosis is often challenging, especially in less-equipped facilities. For instance, due to the nonspecific nature of the symptoms, the diagnosis is often delayed even in well-equipped hospitals. In fact, clinical signs often mimic other infectious, neoplastic, or immune-mediated diseases, leading to late recognition or, in some cases, to a complete lack of diagnosis. Given the systemic nature of the disease, significant multiorgan involvement may be present even when the first clinical sign observed during examination is merely a mild lameness [36].

Accurate diagnosis relies on a combination of tools. As evidenced by the literature review, hematobiochemical analyses often reveal leukocytosis, azotemia, hyperglobulinemia, elevated transaminases, and other markers of systemic inflammation, frequently accompanied by electrolyte imbalances (Table 5), most likely attributable to generic systemic dysfunction. It is clear that the clinical signs and laboratory findings described are highly non-specific and often vague, making them difficult to interpret. Notably, urinalysis is crucial in suspected systemic aspergillosis, as it can reveal signs of renal impairment and, in many cases, hyphae are detectable in urinary sediment, supporting early diagnosis [9,13,15,16,18,21,22,29,32,33,34,38,39,40,51,53].

According to the cases reviewed, diagnostic imaging plays a key role in identifying skeletal abnormalities (e.g., discospondylitis), organ lesions, lymphadenopathy, masses, and effusions (Table 7). Histology can provide additional confirmation, as specific staining techniques allow visualization of fungal elements [13,29,35,47,56]; however, biopsy is not always feasible due to its invasive nature.

Another factor that may lead to underestimation of the actual occurrence of canine systemic aspergillosis could be the lack of culture examination. Among the 50 articles initially selected, four were excluded due to the absence of culture confirmation. Only by isolating *Aspergillus* in culture can the diagnosis of aspergillosis be definitively confirmed. Fungal isolation remains the most important diagnostic step, alongside hematobiochemical testing, imaging, and, when feasible, rapid cytology. As reported in the literature, cultures can be performed primarily from urine sediment and lymph node aspirates, but also from fine-needle aspirates or biopsies of other sites, as well as from body fluids such as thoracic and abdominal effusions, vitreous humor, uterine fluid, or nasal discharge, depending on disease localization (Table 8).

Another finding from the review of the literature is the need for molecular identification of the *Aspergillus* sp. isolated. This is particularly important for isolates belonging to the *Terrei* section, which contains species that are morphologically similar and often indistinguishable from *A. terreus sensu stricto* [53]. Molecular analyses, using specific markers such as β-tubulin and calmodulin, are therefore recommended to achieve a definitive diagnosis [82]. It should be noted, however, that molecular techniques are not always available in small facilities, and often require referral to specialized laboratories for analysis. Nevertheless, in recent years, awareness of the importance of their use appears to be increasing.

Treatment is another critical point, both in terms of owner management and adverse effects for the animals. As described in the literature, Itraconazole is typically the first-line therapy [27]. Other alternatives, such as posaconazole, voriconazole, or amphotericin B, exist but are often limited by prohibitive costs and potential adverse effects [13,45,47,50], which may lead owners to choose itraconazole as the most accessible option, to refuse therapy or to discontinue it. Consistent administration is essential: interruptions or irregular dosing have been associated with higher relapse rates and short-term mortality [13,51].

It should be noted that the studies included are single case reports or small case series, which limits the generalization of the findings. Moreover, follow-up is often incomplete, and standardized diagnostic protocols are lacking.

Reviewing the literature, it can be observed that survival time is highly variable, from 6.5 years [27] to a few weeks after diagnosis [50], depending on the stage of the disease at diagnosis, the owners’ financial ability to provide long-term treatment, and the individual response of each animal. In any case, it should be noted that the life expectancy of affected animals is generally reduced compared to the average expected for the breed.

## 5. Conclusions

Systemic aspergillosis in dogs remains a challenging condition to diagnose and manage, largely due to its non-specific and highly variable clinical presentation and may be more prevalent than the current literature suggests.

This disease should always be considered in the differential diagnosis in cases presenting clinical signs affecting the neurological, musculoskeletal, respiratory, gastrointestinal, ocular, and urogenital systems. Special attention should be given to the German Shepherd dog, which is the most frequently affected breed.

Timely initiation of antifungal therapy, ideally with consistent administration and, when necessary, multimodal regimens, is critical to improving outcomes. However, treatment decisions must balance efficacy, cost, and tolerance. Increased awareness, standardized diagnostic protocols, careful management and more robust clinical studies are needed to advance early recognition and improve prognosis and quality of life in dogs affected by systemic aspergillosis.

## Figures and Tables

**Table 2:** Geographic distribution of reported canine systemic aspergillosis by country.

Country	No. of Publications	References
United States of America	19	[9,15,16,17,20,23,25,29,30,32,34,38,45,49,52,53,55,56,57]
Australia	11	[18,19,21,24,27,31,39,40,41,48,54]
Italy	5	[12,13,37,42,51]
Israel	2	[4,14]
Poland	1	[43]
Portugal	1	[36]
South Africa	1	[23]
South Korea	1	[50]
Thailand	1	[44]
United Kingdom	1	[35]
Japan	1	[47]
Canada	1	[46]
Switzerland	1	[26]
Total number	46	

**Table 3:** Dog breeds reported in the literature as affected by *Aspergillus* spp. systemic infections.

Dog Breed	Number of Reported Cases	References
German Shepherd	105	[4,9,12,15,16,17,18,19,20,21,22,23,24,25,26,27,29,32,33,34,37,39,40,41,49,51,55,56]
Mixed breed	12	[9,13,15,27,36,37,46,53]
Labrador retriever	5	[9,30,44]
Vizsla	3	[15,27]
English Springer spaniel	2	[38,52]
Pug	2	[9,35]
Rhodesian Ridgeback	2	[9]
Newfoundland	1	[15]
Giant Schnauzer	1	[15]
Shiba Inu	1	[47]
Miniature Schnauzer	1	[57]
Saint Bernard	1	[43]
Cocker spaniel	1	[45]
Rottweiler	1	[48]
Coton de Tulear	1	[50]
Dalmatian	1	[19]
Red cloud kelpie	1	[21]
English setter	1	[9]
Whippet	1	[9]
Bull terrier	1	[37]
Flat Coated Retriever	1	[26]
Kurzhaar dog	1	[42]
Not available	10	[27,54]

**Table 4:** Overview of clinical signs organized according to the body systems involved. Bold text indicates the total number of alterations for each body system.

Category	Clinical Sign	No. of Cases	References
GENERAL SIGNS	Weakness/Lethargy	56	[4,12,15,18,19,20,21,22,23,25,27,29,32,34,47,50,52]
	Weight loss	51	[9,15,16,18,19,22,23,26,27,30,39,48,49]
	Fever	40	[4,9,13,16,18,19,20,21,23,25,29,32,38,39,54,56]
	Lymphadenopathy	35	[9,13,15,18,19,23,27,41,45,46,48,55]
	Anorexia	15	[4,9,12,13,20,23,30,44,50]
	Decreased Appetite	10	[15,16,34,39,46,52]
	**Total General Signs**	**207**	
NEUROLOGICAL SYSTEM	Spinal Pain	28	[9,16,21,22,23,29,37,54]
	Paralysis/Paresis	22	[4,9,16,18,19,22,25,27,34,35,49]
	Ataxia/Vestiboular signs	20	[9,15,24,27,32,34]
	Head tilt	10	[4,9,17,34,35,56]
	Nystagmus	7	[4,17,20,32,33,35,55]
	Absence of menace reflex	6	[4,17,34]
	Mental dullness/Depression	6	[9,13,15,17,38,46]
	Proprioceptive deficits	5	[17,22,23,32]
	Paraspinal hyperesthesia	4	[32,34]
	Decreased postural reaction	3	[34]
	Seizure	3	[9,17,34]
	Positional ventral strabism	2	[34]
	Circling	2	[9,34]
	Hyperreflexive patellar reflex	2	[25,49]
	Absent pupillar reflex	2	[25,34]
	Miosis	2	[25,35]
	Anisochoria	1	[4]
	Motor function deficit	1	[49]
	Right facial paralysis	1	[35]
	Coma	1	[24]
	Opisthotonus	1	[24]
	**Total Neurological Signs**	**129**	
MUSCULOSKELETAL SYSTEM	Lameness	54	[9,13,15,17,18,19,21,23,27,34,36,38,39,42,51,52,56]
	Musculoskeletal pain	24	[27]
	Swollen joint	16	[18,19]
	Lumbar pain	14	[4,19,35]
	Back pain	10	[15,18,21,23]
	Neck pain	6	[18,20,22,25,26,51]
	Muscle atrophy	3	[22,23,49]
	Pathological fractures	2	[16,35]
	Extensor muscles rigidity	2	[23,24]
	Muscle tenderness	1	[20]
	Myositis	1	[52]
	Difficulty in jumping	1	[35]
	Pain when attempting to lie/stand	1	[48]
	**Total Musculoskeletal signs**	**135**	
GASTROINTESTINAL SYSTEM	Vomiting	17	[9,13,15,18,20,21,27,30,44,46,52]
	Diarrhea	7	[15,27]
	Hepatosplenomegaly	2	[45,48]
	Oral ulcer	2	[23]
	Hematochezia	1	[30]
	Melena	1	[30]
	Abdominal pain	1	[46]
	Abdominal distension	1	[12]
	Tense abdomen	1	[23]
	**Total Gastrointestinal Signs**	**33**	
OCULAR SIGNS	Uveitis	13	[4,15,16,18,19,20,54,55]
	Vision deficits	5	[9,17,27,34]
	Retinitis/Chorioretinitis	4	[9,16,23]
	Endophtalmitis	2	[18,57]
	High intraocular pressure	2	[4,32]
	Hyphema	2	[9,32]
	Exophtalmos	2	[4,35]
	Conjunctivitis	2	[25,55]
	Panopthalmitis	1	[9]
	Scleral Injection	1	[38]
	Rubeosis	1	[34]
	Ocular discharge	1	[30]
	Vitreous opacity	1	[25]
	**Total Ocular Signs**	**37**	
GENITOURINARY SYSTEM	Polyuria/Polydipsia	18	[4,13,15,21,26,27,38,39,41]
	Pyometra	3	[18,32,33,55]
	Vulvar sero-sanguineous discharge	2	[32,56]
	Testicular induration	1	[43]
	Atrophic epididymis	1	[43]
	Swollen scrotum	1	[23]
	Urinary incontinence	1	[23]
	Cystitis	1	[16]
	Nocturia	1	[41]
	Presence of hyphae in semen	1	[43]
	Enlarged prostate	1	[30]
	Fresh blood from urethral orifice	1	[40]
	**Total Genitourinary Signs**	**32**	
RESPIRATORY SYSTEM	Cough	9	[9,12,27,38,48,53]
	Respiratory distress	3	[9,38,48]
	Tachypnea	3	[30,39,50]
	Epistaxis	1	[34]
	Mucoid nasal discharge	1	[30]
	Serosanguinous nasal discharge	1	[34]
	Crackles	1	[38]
	Decreased respiratory rate	1	[20]
	Dyspnea	1	[44]
	Labored breathing	1	[53]
	**Total Respiratory Signs**	**22**	
CARDIAC SYSTEM	Cardiac murmur	3	[27,54,56]
	Hypertension	2	[24,46]
	Arrythmia	2	[9,27]
	Bradycardia	1	[46]
	Tachycardia	1	[39]
	**Total Cardiac Signs**	**9**	
DERMATOLOGICAL SIGNS	General skin alteration	3	[15,16,22]
	Pruritus	1	[38]
	Chronic inflammatory process involving overlying skin of the affected bone	1	[23]
	**Total Dermatological Signs**	**5**	

**Table 5:** Summary of hematological, biochemical and urinalysis abnormalities observed in affected dogs.

Category	Category	No. of Cases	References
CBC (Hematology)	Leukocytosis	42	[9,12,13,15,16,17,20,22,23,24,37,38,44,46,50,53,55]
	Neutrophilia	35	[13,15,16,17,18,20,22,23,25,27,35,37,44,46,48,50,53,56]
	Anemia	16	[9,15,27,45,46,48,53,54,56]
	Monocytosis	14	[15,16,17,20,23,35,37,44,46,50,53]
	Eosinophilia	13	[15,16,22,27,32,41]
	Lymphocytosis	9	[27,41,56]
	Lymphopenia	5	[17,18,23,25,44]
	Thrombocytopenia	1	[15]
	Thrombocytosis	1	[12]
Biochemistry	Azotemia	32	[4,13,15,18,20,21,23,25,26,27,30,32,35,39,40,41,46,47,48]
	Hyperglobulinemia	28	[15,22,27,30,32,38,39,41,42,44,46,48]
	Increased ALP activity	19	[4,12,15,18,23,25,27,38,44,45,48,53]
	Increased TSP	13	[4,13,18,24,32,35,40,46]
	Hyperphosphatemia	10	[15,21,26,27,30]
	Increased AST activity	9	[12,15,21,23,25,48]
	Increased ALT activity	9	[12,15,21,25,27,42,48,53]
	Hypoalbuminemia	8	[15,27,38,48]
	Increased CRP	8	[13,37,47,48,50,51]
	Hypercalcemia	4	[21,27]
	Hypocalcemia	3	[27,53]
	Increased CK activity	3	[15,23,25]
	Hyperalbuminemia	2	[27]
	Hyperbilirubinemia	2	[15,53]
	Hypokaliemia	2	[15,53]
	Increased LDH	2	[4,23]
	Hypochloremia	2	[30,46]
	Hyperkaliemia	2	[26,30]
	Hyperfibrinogemia	1	[39]
	Increased amylase	1	[18]
	Hyponatriemia	1	[30]
	Hypoglycemia	1	[41]
	Increased GGT	1	[53]
	Hyperglycemia	1	[53]
Urinalysis	Pyuria	28	[9,13,15,27,32]
	Fungal hyphae in urinary sediment	27	[9,13,15,16,18,21,22,29,32,33,34,38,39,40,51,53]
	Isosthenuria or hyposthenuria	25	[13,15,20,21,23,25,26,27,29,30,32,39,41,53]
	Hematuria	20	[9,15,30,32,48]
	Proteinuria	3	[30,44,48]
	Leukocytes in urine	2	[16,39]
	Bilirubinuria	1	[44]

Table 5 legend: CBC = Complete blood Count; ALP = Alkaline Phosphatase; TSP = Total Serum protein; AST = Aspartate Aminotransferase; ALT = Alanine Aminotransferase; CRP = C-Reactive Protein; CK = Creatine Kinase; LDH = Lactate Dehydrogenase; GGT = Gamma-Glutamyl Transferase.

**Table 6:** Affected portions of the vertebral column in discospondilytis cases. In [9,15], this type of information was not available.

References	No. of Cases	Spinal Region Affected
Walker et al. [56]	1	C4–5, T3–4, T4–5, T5–6, T8–9
Del Magno et al. [13]	1	L1–2
Bruchim et al. [4]	1	L1–2
Day et al. [18]	3	T2–4, T7–8; T5–6, T10–11 C3–4, T11–12 T13–L1
Berry et al. [22]	2	C2–3, C7–11, T2–L1, L2–3, L5–6 T12–13, L5–6, L6–7
Dallman et al. [29]	1	T3–8, T12–13, L3–4, L5–6
Wood et al. [16]	1	T5–6, T8–9, T9–10
Kaufman et al. [32]	1	L7–S1, S2–3, T4–5, T6–7, T8–9, T9–10
Jang et al. [23]	1	L4–5
Thoma et al. [26]	1	C1–2
Okonji et al. [37]	7	T3–L3 T3–L3 T3–L3 T3–L3 T3–L3 T3–L3 T3–L3
Bordoni et al. [51]	1	C6–7, C7–T1, T5–9, T10–11, T12–13
Taylor et al. [34]	1	C5–6, T11–12, T12–13, L5–6, L6–7, L7–S1
Brocal et al. [35]	1	L1–2, L2–3, L3–4, L4–5, L5–6
Zhang et al. [49]	1	T9–10
Gelatt et al. [25]	1	T7–8
Kelly et al. [21]	2	L3–4, T7–8, T11–12, T13–L1 T1–2, T2–3, T6–7, T11–12, L1–2, L2–3, L3–4, L4–5
Bennett et al. [41]	1	T2–3, T4–5

**Table 7:** Summary of the main imaging findings observed on radiography, MRI, CT, and ultrasonography.

Imaging Modality	Findings	No. of Cases	References
Radiography (X-ray)	Discospondylitis	51	[4,9,15,16,18,21,22,25,32,37,41,49,51]
	Osteomyelitis	16	[4,9,15,21,36,49]
	Destructive bone lesion and periosteal reaction	16	[4,13,18,20,21,23,26,30,34,36,39,56]
	Lymph node enlargement	15	[9,12,23,38,44,50,56]
	Vertebral endplate lysis	9	[9,22,32,35,49]
	Mediastinal mass	8	[9,23,39,44,46,50]
	Pulmonary involvement	7	[9,15]
	Pleural effusion	7	[9,12,20,38]
	Large uterus	2	[18,32]
	Abdominal effusion	1	[52]
	Splenomegaly	1	[23]
	Hepatomegaly	1	[23]
	Degenerative joint disease	1	[38]
	Intervertebral disk collapse	1	[56]
	Increased airway and vascular density	1	[16]
	Atelectatis	1	[42]
	Interstitial-nodular pattern	1	[42]
	Cardiomegaly	1	[16]
	Large kidney	1	[18]
	Gluteal muscle enthesitis	1	[13]
	Microcardia	1	[30]
Ultrasound (US)	Kidney and urinary tract involvement	28	[9,13,15,41]
	Splenic involvement	21	[9,12,15,41,45,46]
	Abdominal lymph node enlargement	19	[9,15,41,45,50]
	Hepatic involvement	2	[9,12,45]
	Thickened gastric walls	2	[9]
	Ascites	2	[9]
	Venous thrombi	2	[9]
	Thoracic lymph node enlargement	1	[50]
	Enlarged uterus	1	[56]
	Pancreatic lobe enlargement	1	[9,44]
	Peritoneal effusion	1	[12]
	Iliac lymphadenoegaly	1	[12]
	Abdominal mass	1	[46]
	Cardiomegaly	1	[16]
	Abdominal effusion	1	[52]
	Pleural effusion	1	[53]
	Pericadial effusion	1	[53]
Computed Tomography (CT)	Discospondylitis	7	[37]
	Enlargement of thoracic and abdominal lymph nodes	4	[9,44,48,50]
	Enlargement of iliac lymph node	1	[47]
	Lysis of vertebral endplates	1	[41]
	Hepatomegaly	1	[48]
	Splenomegaly	1	[48]
	Bronchial pattern	1	[48]
	Kidneys calcification	1	[41]
	Lytic bone lesion	1	[35]
Magnetic Resonance Imaging (RMI)	Discospondylitis	9	[34,37,51]
	Brain lesions	3	[9]
	Bone lysis	2	[13,35]
	Periosteal proliferation	2	[13,36]
	Enlargement of lymph nodes	1	[13]
	Muscular lesion	1	[13]

**Table 8:** Isolation sources for fungal culture used in the studies analyzed.

Isolation Source	No. of Cases	References
Culture of urine	65	[4,9,13,15,18,21,22,24,27,29,32,33,34,38,40,51]
Culture from organs after necropsy	33	[4,9,12,13,18,19,33,34,38,39,49,53]
Culture from FNA of organs	20	[9,27,44,57]
Culture from fine needle aspiration (FNA) of lymph node	9	[9,13,41,46]
Culture of bone biopsy	6	[9,29,35,36,56]
Culture of lymph node biopsy	5	[38,47,48,55]
Culture of intervertebral space aspirate	5	[9,18,22]
Blood culture	5	[9]
Culture of joint fluid	3	[9,18]
Culture of CSF	3	[9,34]
Culture of pleural effusion	3	[9]
Culture of curettage of intervertebral disc	2	[22,35]
Cultured swab sample taken during surgery/surgical sites	2	[34,50]
Culture of abdominal effusion	1	[52]
Culture of vitreous centesis	1	[25]
Culture from surgical biopsy	1	[50]
Culture from spinal mass	1	[15]
Culture of fluid from a cystic renal mass	1	[15]
Culture of nasal biopsy	1	[34]
Culture of BAL	1	[42]
Culture of aspirate bone lesion	1	[18]
Culture of aspirate hock lesion	1	[18]
Culture of uterine fluid	1	[56]
Culture of bone marrow	1	[45]
Not available	3	[19,54]

**Table 9:** Organs exhibiting macroscopic alterations at necropsy.

Organ Exhibiting Macroscopic Alterations at Necropsy	No. of Cases	References
Kidney	55	[4,9,13,16,17,18,19,20,21,22,23,24,25,26,29,32,34,38,39,49,52,53,55]
Spleen	43	[4,9,16,17,18,19,20,21,22,23,24,25,26,32,39,52,55]
Vertebrae and bones	40	[9,13,18,19,20,21,22,23,29,39,49]
Lymph nodes	32	[4,9,13,16,19,20,22,23,32,34,38,39]
Heart (myocardium, pericardium, endocardium)	30	[4,9,16,17,19,20,22,25,38,39,52,53]
Liver	16	[9,16,17,20,23,25,38,52]
Muscle	13	[19,20,23,25,38,52]
Brain	12	[4,9,17,20,23,24,32,34]
Lung	9	[9,16,17,23,55]
Pancreas	8	[9,16,20,23,38]
Eye	8	[9,16,20,25,55]
Bone marrow	6	[9,16,19]
Diaphragm	4	[20,25,34,52]
Pleura	4	[9,23,38,52]
Stomach	4	[23,25,52]
Spinal cord	3	[9,23,25]
Uterus	3	[16,32,33]
Joint	3	[16,38]
Meninges	2	[9,22]
Adrenal glands	2	[38,39]
Small intestine	2	[9,23]
Periarticular tissue	1	[16]
Cauda equina	1	[29]
Peritoneum	1	[52]
Gallbladder	1	[52]
Skin	1	[9]
Prostate	1	[9]
Urinary bladder	1	[26]
Testis	1	[24]
Trachea/larynx	1	[9]

## Data Availability

No new data were created or analyzed in this study.
